# Phenylmethimazole Suppresses dsRNA-Induced Cytotoxicity and Inflammatory Cytokines in Murine Pancreatic Beta Cells and Blocks Viral Acceleration of Type 1 Diabetes in NOD Mice

**DOI:** 10.3390/molecules18043841

**Published:** 2013-03-27

**Authors:** Kelly D. McCall, Martin J. Schmerr, Jean R. Thuma, Calvin B. L. James, Maria C. Courreges, Fabian Benencia, Ramiro Malgor, Frank L. Schwartz

**Affiliations:** 1 Department of Specialty Medicine, Ohio University Heritage College of Osteopathic Medicine, Athens, OH 45701, USA; 2 Department of Biomedical Sciences, Ohio University Heritage College of Osteopathic Medicine, Athens, OH 45701, USA; 3 The Diabetes Institute, Ohio University Heritage College of Osteopathic Medicine, Athens, OH 45701, USA; 4 Department of Biological Sciences, Ohio University College of Arts & Sciences, Athens, OH 45701, USA; 5 Molecular & Cellular Biology Program, Ohio University College of Arts & Sciences, Athens, OH 45701, USA; 6 Biomedical Engineering Program, Ohio University Russ College of Engineering & Technology, Athens, OH 45701, USA; 7 Department of Natural Sciences, Central Ohio Technical College Newark, OH 43055, USA

**Keywords:** type 1diabetes, dsRNA, beta cell death, phenylmethimazole (C10)

## Abstract

Accumulating evidence supports a role for viruses in the pathogenesis of type 1 diabetes mellitus (T1DM). Activation of dsRNA-sensing pathways by viral dsRNA induces the production of inflammatory cytokines and chemokines that trigger beta cell apoptosis, insulitis, and autoimmune-mediated beta cell destruction. This study was designed to evaluate and describe potential protective effects of phenylmethimazole (C10), a small molecule which blocks dsRNA-mediated signaling, on preventing dsRNA activation of beta cell apoptosis and the inflammatory pathways important in the pathogenesis of T1DM. We first investigated the biological effects of C10, on dsRNA-treated pancreatic beta cells in culture. Cell viability assays, quantitative real-time PCR, and ELISAs were utilized to evaluate the effects of C10 on dsRNA-induced beta cell cytotoxicity and cytokine/chemokine production in murine pancreatic beta cells in culture. We found that C10 significantly impairs dsRNA-induced beta cell cytotoxicity and up-regulation of cytokines and chemokines involved in the pathogenesis of T1DM, which prompted us to evaluate C10 effects on viral acceleration of T1DM in NOD mice. C10 significantly inhibited viral acceleration of T1DM in NOD mice. These findings demonstrate that C10 (1) possesses novel beta cell protective activity which may have potential clinical relevance in T1DM and (2) may be a useful tool in achieving a better understanding of the role that dsRNA-mediated responses play in the pathogenesis of T1DM.

## 1. Introduction

Accumulating evidence supports a role for viruses in the pathogenesis of type 1 diabetes mellitus (T1DM) [1,2,3,4,5,6,7,8,9,10,11,12,13,14,15,16,17,18,19,20]. Viral infection of beta cells can initiate disease by direct cell damage, beta cell toxicity from the acute innate antiviral response, or release/induction of endogenous beta cell self-antigens triggering autoimmune destruction [11,13,14,21,22]. Double-stranded RNA (dsRNA) is a molecular intermediate formed during the replication cycle of many viruses. Activation of dsRNA-sensing pathways such as TLR3 and RIG1 by dsRNA leads to beta cell apoptosis and expression of pro-inflammatory genes known to be important in the development of T1DM [23,24]. Polyinosinic-polycytidylic acid (pIC) is a synthetic dsRNA that mimics host responses triggered by viral dsRNA, including direct viral toxicity and antiviral responses that can trigger autoimmune mechanisms. For example, pIC induces apoptosis of beta cells and impairs beta cell function [24]. High doses of pIC precipitated diabetes in both diabetes-prone BB (DP-BB) and diabetes-resistant (DR-BB) rats [25,26] and accelerated the onset of T1DM in non-obese diabetic (NOD) mice [27]. In addition, pIC has been shown to induce insulitis and accelerate the progression to T1DM in RIP B7.1 mice [28], and induce insulitis, but not T1DM, in BALB/c mice [29]. Type 1 interferons (specifically, IFNα and IFNβ), which are strongly induced by pIC, have been associated with the pathogenesis of T1DM [24,30,31,32,33], and their therapeutic use in diseases, such as chronic active hepatitis, has triggered autoimmune diseases including T1DM [34]. These type 1 interferons have been shown to accelerate the onset of diabetes in NOD mice, induce insulitis in diabetes resistant strains [35], and are present in the beta cells of recent-onset T1DM patients [31,36]. pIC also induces other inflammatory mediators that exacerbate the autoimmune process, including CXCL10 and major histocompatibility (MHC) Class I and II molecules [37,38]. 

We have previously shown that phenylmethimazole (C10), a small molecule derivative of methimazole, effectively blocks dsRNA induction of co-stimulatory and adhesion molecules, chemokines, pro-inflammatory cytokines and their receptors, and MHC gene expression in thyrocytes, pancreatic cancer cells, and melanoma cells [37,38,39]. More recently, we demonstrated that C10 blocks dsRNA-induced IRF3 homodimer formation in pancreatic ductal epithelioid carcinoma cells [40], an important step in the TLR3 and RIG1 signaling pathways [23,41]. This suggests that C10 may have biological activities that protect pancreatic beta cells from dsRNA-induced beta cell death and upregulation of many cytokines and chemokines involved in the pathogenesis of T1DM. Thus, these studies were designed to evaluate and describe potential beneficial biological effects C10 may have on preventing dsRNA activation of beta cell apoptosis and inflammatory pathways important in the development of T1DM as well as to evaluate efficacy of C10 to block viral acceleration of T1DM *in vivo*. 

## 2. Results and Discussion

### 2.1. C10 Protects Pancreatic Beta Cells from dsRNA-Induced Cytotoxicity

In order to study the effects of C10 on dsRNA-induced beta cell cytotoxicity, we utilized two mouse pancreatic beta cell lines, TC-6 and NIT-1, in culture. NIT-1 cells are derived from the NOD mouse [42], which is genetically prone to spontaneously develop autoimmune-mediated T1DM, whereas TC-6 cells are derived from transgenic mice that do not spontaneously develop autoimmune-mediated T1DM [43,44]. Thus, the inflammatory response to dsRNA in NIT-1 cells is expected to be most representative of the inflammatory response to dsRNA that occurs *in vivo* in the NOD mouse model, which triggers an autoimmune-mediated (Pattern A) form of T1DM [20,45,46]. However, the effects of dsRNA on TC-6 beta cell viability is expected to be most representative of the direct viral cytotoxicity seen in the Pattern B form of T1DM [20,45,46].

**Figure 1:**
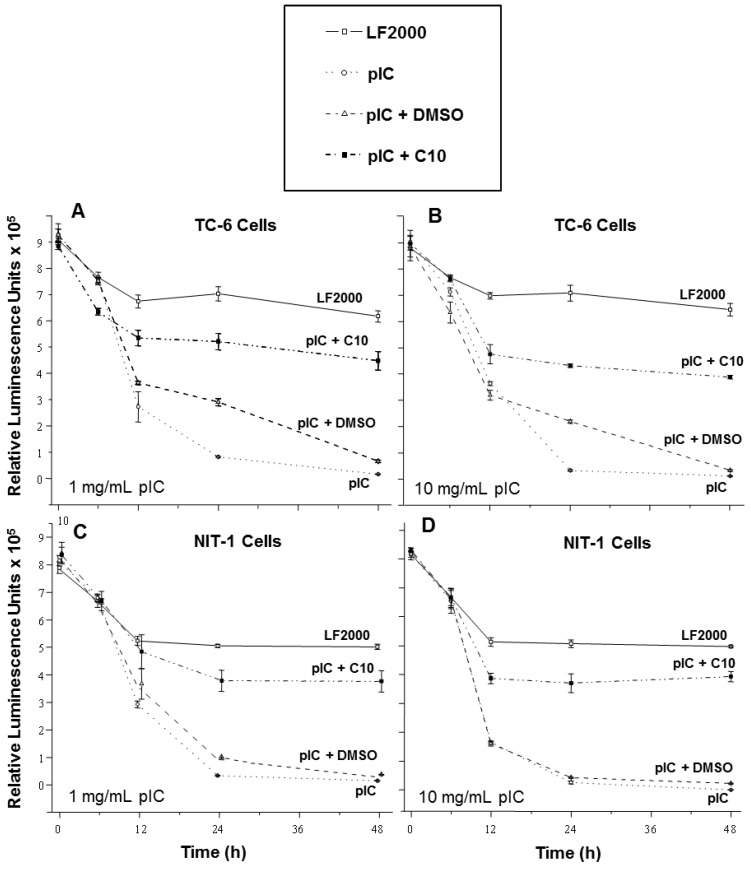
C10 prevents pIC-induced cytotoxicity in pancreatic beta cells in culture. TC-6 and NIT-1 cells were transfected with 1 mg/mL (**A** & **C**) and 10 mg/mL (**B** & **D**) of pIC. TC-6 and NIT-1 cells were either mock transfected (— □ —), transfected with pIC (^…^ ○ ^…^), or transfected with pIC and treated with either DMSO (solvent)(– – Δ – –) or 0.5 mM C10 (– · ■ – ·) for the indicated times. At 6, 12, 24, and 48 h post-transfection, the viability of cells was measured using the Cell Titer-Glo Luminescent Cell Viability Assay.

Transfection of TC-6 cells with a low concentration of pIC (1 μg/mL) steadily decreased cellular viability over a 48-hour period (Figure 1A) while higher concentrations of pIC (10 μg/mL) did not further increase this cytotoxic effect (Figure 1B). Treatment with C10 significantly diminished the cytotoxic effects of pIC transfection with either low (Figure 1A) or high concentrations (Figure 1B), although it was not as protective at the higher concentration of pIC. The solvent used for dissolving C10 (a solution containing 0.25% DMSO) provided no protection of TC-6 cells transfected with either concentration of pIC except at the 24-hour time point where it provided only minimal protection (Figure 1A,B).

Similar results were obtained in NIT-1 cells. Transfection of NIT-1 cells with pIC (1 μg/mL) also reduced cell viability in a dose-dependent manner (Figure 1C,D) with higher concentrations (10 μg/mL) inducing greater cytotoxicity (Figure 1D). NIT-1 cells treated with C10 following pIC transfection (Figure 1C,D) were protected from the cytotoxic effects of pIC at both concentrations (Figure 1C,D) similar to that seen with the TC-6 cells, while the solvent had no effect on NIT-1 cell viability.

These data are consistent with previous studies demonstrating the cytotoxic effect of pIC in pancreatic beta cells [24] and show for the first time that C10 suppresses the acute induction of beta cell toxicity in response to dsRNA in both transfected beta cell lines. The cytotoxic effect of transfection with pIC on the NIT-1 cell line was concentration-dependent, while the TC-6 cell line was much more sensitive to the pIC treatment because the low dose rapidly induced cytotoxicity. This result contrasts with the observation of Robbins* et al.* who reported that higher concentrations of pIC did not increase cytotoxicity in NIT-1 cells [47]. One explanation for this discrepancy may be that the longer exposure of cells (48 h) to pIC-liposome complexes in our studies results in the activation of additional factors involved in programmed cell-death pathways.

In sum, C10 suppresses the cytotoxic effects of dsRNA on both transfected beta cell lines, suggesting that C10 may prevent viral induction of beta cell death seen in both Pattern A and B forms of T1DM.

### 2.2. C10 Blocks dsRNA-Induced Upregulation of TLR3 Expression and Signaling Products in Pancreatic Beta Cells

**Figure 2:**
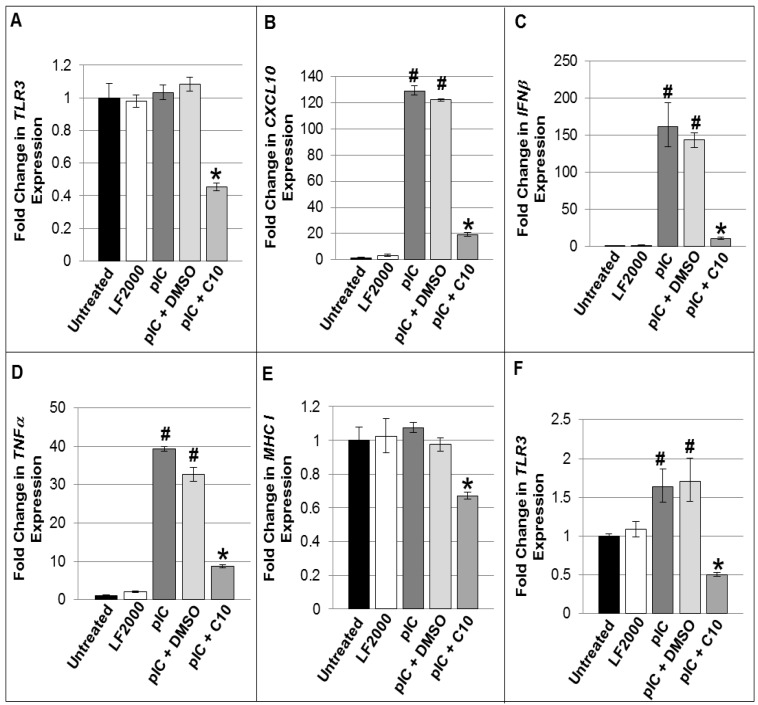
C10 suppresses pIC-induced expression of TLR3 and of other genes involved in the inflammatory response in NIT-1 cells. Quantitative Real Time PCR was used to characterize the expression of TLR3 and cytokines and chemokines involved in mediating insulits and type 1 diabetes. Total RNA was isolated from NIT-1 cells transfected with 1 μg/mL of pIC using Lipofectamine 2000 (LF2000) plus DMSO (solvent) or 0.5 mM C10 for 4 h (**A**–**E**), or 6 h (**F**) and gene expression was evaluated using the murine TLR3, CXCL10, IFNβ, TNFα, and MHC Class I Gene Expression Assays (Applied Biosystems, Carlsbad, CA). Fold changes in gene expression were calculated using the ΔΔCt method. Statistical analyses were performed using one-way ANOVAs followed by Bonferroni and Tukey-Kramer post-hoc analyses. (**A**) * Indicates a significant reduction in TLR3 gene expression by C10, *p* < 0.00001; (**B**–**D**) # Indicates a significant induction of gene expression by pIC, *p* < 0.00001, and * indicates a significant reduction in pIC-stimulated gene expression by C10, *p* < 0.00001; (**E**) * indicates a significant reduction in basal MHC Class I expression, *p* < 0.001; and in (**F**) # Indicates a significant induction of TLR3 expression by pIC, *p* < 0.05, and * indicates a significant reduction in pIC-stimulated TLR3 expression by C10, *p* < 0.05.

It is hypothesized that the pancreatic beta cell itself is an important source of the pro-inflammatory cytokines that mediate beta cell apoptosis, as well as expression/release of intracellular auto-antigens propagating the autoimmune-mediated beta cell destruction. Furthermore, dsRNA activation of dsRNA-sensing pathways, such as TLR3, triggers the production of these pro-inflammatory cytokines and chemokines in beta cells [23]. Since we have previously shown that C10 is a potent inhibitor of dsRNA-induction of the same pro-inflammatory cytokines and chemokines involved in the development of T1DM (CXCL10, IFNβ, TNFα, TLR3, and MHC Class I) in other non-immune cell types [37,38,39,40], we hypothesized that C10 may also be an important and potent inhibitor of dsRNA-induced production of these cytokines and chemokines in pancreatic beta cells. To test this hypothesis, we transfected NIT-1 cells with pIC in the presence or absence of C10 and evaluated CXCL10, IFNβ, TNFα, TLR3, and MHC Class I gene expression. Comparative multiplex real-time PCR was used to characterize the effects of pIC transfection on the expression of TLR3 and TLR3 signaling products and to determine the effect of C10 or its vehicle (DMSO) in NIT-1 cells in culture. While after 4 h there was no induction of TLR3 gene expression by pIC transfection (Figure 2A), however, by 6 h post-transfection, induction of TLR3 gene expression was evident (Figure 2F). Additionally, while there was no stimulation of TLR3 by 4 h post-pIC treatment, none-the-less, C10 suppressed TLR3 below basal expression following treatment at this time point (Figure 2A), and prevented pIC upregulation of TLR3 gene expression at 6 h post pIC treatment (Figure 2F). pIC transfection also significantly increased CXCL10, IFNβ, and TNFα gene expression while C10 significantly blocked pIC stimulation of CXCL10, IFNβ, and TNFα expression (Figure 2B–D). Similar to its effect on TLR3 expression, C10 also significantly reduced basal MHC Class I gene expression (Figure 2E). Although pIC did not up-regulate MHC Class I expression above basal levels, C10 did significantly reduce its expression below basal levels, suggesting that C10 may be blocking basally-activated signaling pathways that control its expression. C10 inhibition of pIC-induced TLR3 expression and downstream signaling molecules in NIT-1 cells was additionally confirmed by quantitative real-time PCR arrays and similar findings were also obtained in TC-6 cells (Table 1, Table 2 and Table 3).

**Figure 3:**
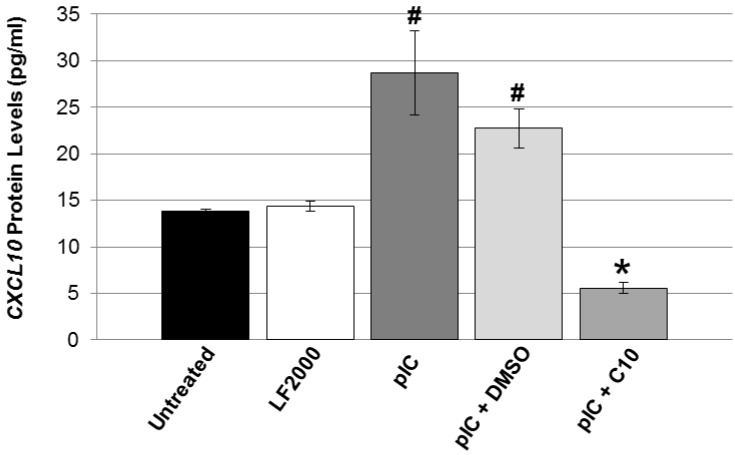
C10 blocks pIC-stimulated CXCL10 protein levels. CXCL10 protein levels were measured in the cell culture supernatants from NIT-1 cells transfected with 1 μg/mL of pIC plus DMSO (solvent) or 0.5 mM C10 for 4 h using the mouse CXCL10/IP-10 Quantikine ELISA kit from R & D Systems. Statistical analysis was performed using a one-way ANOVA followed by Bonferroni and Tukey-Kramer post-hoc analysis. # Indicates a significant induction of CXCL10 protein levels by pIC, *p* < 0.05, and * indicates a significant reduction in pIC-stimulated CXCL10 protein levels by C10, *p* < 0.05.

In agreement with our gene expression studies above, we observed a significant increase in CXCL10 protein levels following pIC transfection and significant inhibition of pIC-induced CXCL10 protein levels with C10 treatment (Figure 3). IFNβ and CXCL10 are thought to be key players in the pathogenesis of T1DM. As previously mentioned, type 1 interferons (IFNα and IFNβ) have been associated with the pathogenesis of type 1 diabetes [24,30,31,32,33,34,35,36,48], and their therapeutic use in diseases such as chronic active hepatitis has triggered autoimmune diseases including type 1 diabetes [34]. These type 1 interferons accelerate the onset of diabetes in NOD mice [35], induce insulitis in diabetes resistant strains [35], and are present in the beta cells of recent-onset diabetic patients [31,36]. CXCL10 expression in NOD mice is responsible for the targeted migration of autoreactive T-cells into the pancreatic islets [49]. Furthermore, it has been shown that notably high levels of CXCL10, followed by CCL2 and CCL5 [50], are constitutively expressed from pancreatic islets, suggesting that beta cell production of these chemokines is important in the development of T1DM. 

**Table 1:** Relative fold changes induced by pIC and inhibition by C10 on TLR signaling array.

Functional Gene Groupings/Gene Name				TC-6 cells			NIT-1 Cells		
**Pattern Recognition Receptors**			pIC	pIC + DMSO	pIC + C10	pIC		pIC + DMSO	pIC + C10
Toll-Like Receptor 1			5.78	<5	<5	<5		<5	<5
Toll-Like Receptor 2			8.20	<5	<5	26.17		11.59	<5
Toll-Like Receptor 3			66.7	23.2	<5	176.0		64.2	<5
Toll-Like Receptor 4			5.78	<5	<5	<5		<5	<5
Toll-Like Receptor 6			<5	<5	<5	22.24		<5	<5
Toll-Like Receptor 7			5.74	<5	<5	<5		<5	<5
Toll-Like Receptor 8			5.74	<5	<5	<5		<5	<5

**Table 2:** Relative fold changes induced by pIC and inhibition by C10 on expression of genes in a TLR signaling array.

Functional Gene Groupings/Gene Name		TC-6 cells		NIT-1 Cells		
	pIC	pIC + DMSO	pIC + C10	pIC	pIC + DMSO	pIC + C10
**TLR adaptor proteins**						
Heat shock 70kDa protein 1A (Hspa1a)	<5	<5	<5	20.32	<5	8.30
TICAM2	<5	<5	<5	9.58	6.57	<5
**Transcription factors**						
c-Fos	<5	<5	<5	5.45	<5	<5
c-Jun	<5	<5	<5	14.37	<5	<5
CCAAT/enhancer binding protein β (Cebpb)	<5	<5	<5	9.22	<5	<5
**NF-κB pathway**						
CCL2	21.50	22.25	<5	172.45	30.70	<5
CD80	6.03	6.69	<5	40.22	20.97	<5
CD86	<5	<5	<5	68.12	35.38	<5
Granulocyte macrophage colony-stimulating factor (Csf2)	<5	<5	<5	5.92	<5	<5
Granulocyte colony-stimulating factor (Csf3)	<5	<5	<5	6.80	<5	<5
IKKα (Chuk)	<5	<5	<5	5.37	<5	<5
IL-1α	<5	<5	<5	7.31	<5	<5
IL-2	5.78	<5	<5	<5	<5	<5
IL-6	8.91	8.98	<5	580.04	143.01	<5
IL-6 Receptor α (IL-6ra)	<5	<5	<5	25.46	<5	<5
IL-10	<5	<5	<5	57.68	22.24	<5
IL-12A	6.62	<5	<5	10.78	22.09	<5
NF-κB2	8.52	<5	<5	22.86	16.56	<5
IκBα (Nfkbia)	<5	<5	<5	13.50	<5	<5
IκBβ (Nfkbib)	<5	<5	<5	14.47	<5	<5
Prostaglandin-endoperoxide synthase 2 (Ptgs2)	<5	<5	<5	48.34	17.69	<5
TNF-α	5.78	<5	<5	26.72	6.70	<5
TNF-β (Lta)	NS	<5	<5	45.57	38.59	<5
Tumor necrosis factor, alpha-induced protein 3 (Tnfaip3)	7.87	<5	<5	<5	<5	<5
**IRF pathway**						
IP-10 (CXCL10)	189.49	74.85	<5	543.07	141.53	<5
IFN-β1	135.86	72.81	<5	2778.33	196.04	<5
IRF-1	6.85	6.32	<5	12.04	11.24	<5

NS = > 5-fold, but not statistically significant *p* > 0.05.

**Table 3:** Relative fold changes induced by pIC and inhibition by C10 on expression of inflammatory cytokine gene array.

Functional Gene Groupings/Gene Name		TC-6 Cells				NIT-1 Cells		
		pIC	pIC + DMSO	pIC + C10	pIC		pIC + DMSO	pIC + C10
**Chemokines**								
CCL2		13.60	6.96	<5	160.12		25.05	<5
CCL4		39.15	5.94	<5	25.33		25.58	<5
CCL5		320.9	112.21	<5	1245.92		384.54	<5
CCL7		18.2	14.42	<5	107.49		91.90	<5
CCL9		<5	<5	<5	6.86		<5	<5
CCL12		8.76	<5	<5	19.95		8.80	<5
CCL19		<5	<5	<5	26.32		32.38	64.40
CCL20		183.67	54.19	<5	76.27		43.17	<5
CCL25		23.36	21.33	<5	<5		<5	<5
CXCL1		7.57	<5	<5	<5		<5	<5
CXCL5		5.86	<5	<5	<5		10.14	<5
CXCL9		58.32	17.94	<5	344.41		275.71	<5
CXCL10		150.23	133.9	<5	522.03		170.31	<5
CXCL11		<5	<5	<5	74.70		67.51	<5
**Chemokine receptors**								
CCR10		179.27	57.48	<5	<5		<5	<5
**Cytokines**								
IL-1F6		<5	<5	<5	38.94		15.97	<5
IL-4		41.53	11.84	<5	<5		<5	<5
IL-10		<5	<5	<5	28.11		10.87	<5
IL-11		33.61	10.67	<5	19.00		8.65	<5
IL-15		139.68	43.26	<5	36.45		35.19	<5
Osteopontin (Spp1)		18.33	NS	<5	<5		<5	<5
TGF-β1		62.51	20.32	<5	<5		<5	<5
TNF-α		<5	<5	<5	21.38		5.76	<5
TNF-β (Lta)		<5	<5	<5	38.27		34.82	<5
Lymphotoxin-β (Ltb)		299.41	13.83	<5	<5		<5	<5
**Cytokine receptors**								
Glycoprotein 130 (IL-6st)		324.26	110.66	<5	<5		<5	<5
IL-1R2		21.27	5.82	<5	<5		<5	<5
IL-5Rα		11.88	<5	<5	<5		<5	<5
Tumor necrosis factor receptor superfamily 1B (Tnfrsf1b)		−23.65	−62.47	<5	<5		<5	<5
Integrin β2 (Itgb)		<5	<5	<5	10.54		<5	<5
**Other inflammatory genes**								
Caspase 1 (Casp1)		17.52	6.06	<5	15.98		7.02	−6.15

NS = > 5-fold, but not statistically significant *p* > 0.05.

Elevated serum levels of CXCL10 have also been observed in patients with T1DM as well as in islet-cell autoantibody positive individuals who are at risk for developing T1DM [51,52]. Pathogenic Th1 cells predominately express CXCR3, the receptor for CXCL10, suggesting that CXCL10 accelerates the autoimmune process by promoting the migration of autoreactive Th1 cells to the pancreas [53]. In our current studies pIC strongly induced the expression of IFNβ and CXCL10 in NIT-1 cells by over 120 fold, while C10 completely suppressed pIC-induced expression of both genes (IFNβ and CXCL10). Together, the fact that these genes are critical to the development of type 1 diabetes and that C10 dramatically reduces pIC induction of these genes in beta cells further supports the idea that C10 may be useful for the prevention of viral-induced type 1 diabetes (both pattern A and pattern B). Presently, the major barrier to such use is the lack of sensitivity and specificity in identifying those individuals at risk [54].

### 2.3. C10 Attenuates Coxsackievirus B4 (CVB4) Acceleration of T1DM in Non-Obese Diabetic (NOD) Mice

**Figure 4:**
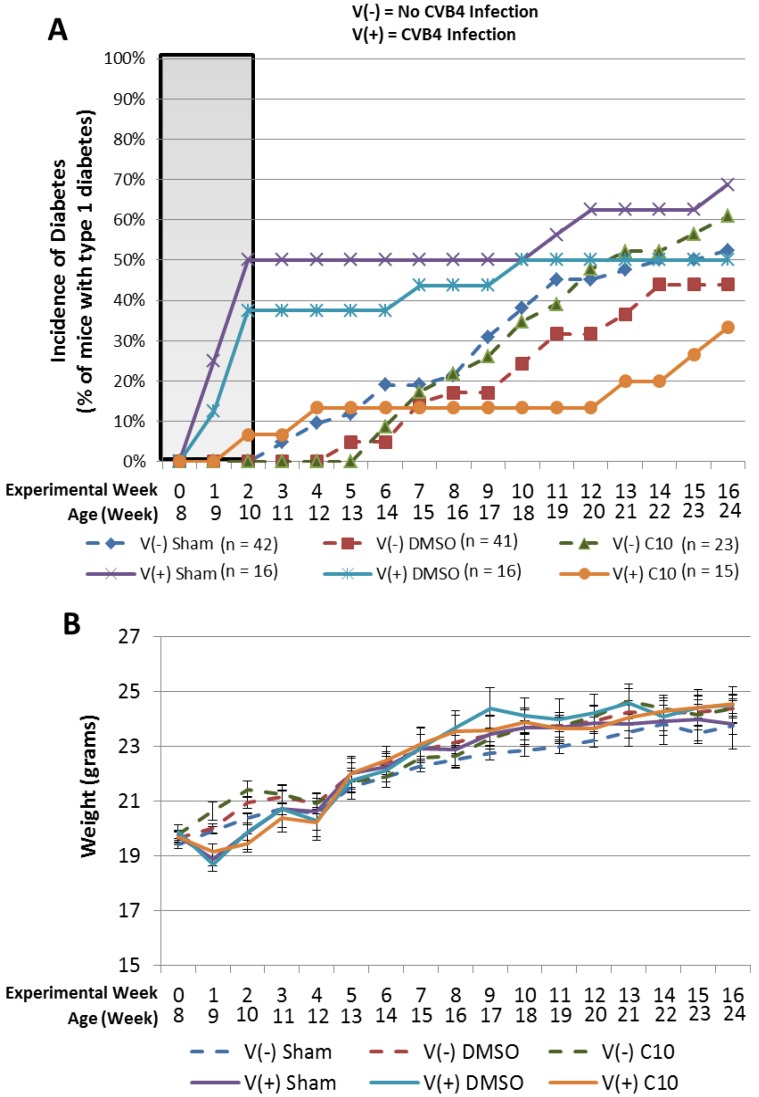
C10 attenuates CVB4 acceleration of type 1 diabetes in NOD mice without toxicity. Eight week old female mice were either infected intraperitoneally with 500,000 pfu of CVB4 or mock infected and/or treated with 1 mg/kg C10, DMSO (solvent control), or sham injection (stress control) as indicated. Incidence of diabetes and weights of mice in each group were recorded weekly. (**A**) Effect of indicated treatment on incidence of diabetes in NOD mice infected with CVB4 (solid lines) or uninfected (dashed lines). (**B**) Effect of indicated treatment on weight of NOD mice.

Since C10 significantly blocked dsRNA induction of beta cell death and upregulation of cytokines and chemokines important in the development of T1DM, we investigated the possibility that C10 may block viral induction of T1DM *in vivo*. To test the hypothesis that C10 may be useful to block viral induction of type 1 diabetes, we utilized the coxsackievirus B4 (CVB4)-induced NOD mouse model of type 1 diabetes [55]. Acceleration of T1DM in NOD mice by CVB4 can only be achieved once a critical mass of autoreactive T cells is present in pancreatic islets [55]. If this critical mass of autoreactive T cells is not present in pancreatic islets prior to infection, then CVB4 infection provides long-term protection from disease expression [55]. Serreze *et al.* previously established that once NOD mice have accumulated a naturally occurring critical mass of pancreatic islet autoreactive T cells, which occurs in most NOD mice by 8 weeks of age, a significant acceleration of T1DM can be observed following inoculation with CVB4 [55]. Thus, we infected 8 week old NOD mice with CVB4 as previously described [55] and observed the incidence of diabetes over a 16 week period. For this experiment, groups of mice (infected or un-infected controls) were treated with a sham injection (intraperitoneal needle stick only) as a stress control, or were treated once daily with intraperitoneal injections of DMSO (solvent) or C10 (1 mg/kg). Consistent with previous observations [55], CVB4 infection of 8 week old NOD mice resulted in a rapid acceleration of diabetes, with 50% of mice becoming diabetic by 2 weeks post CVB4 infection (10 weeks of age) (Figure 4A, shaded region). Similar results were observed in DMSO-treated NOD mice infected with CVB4, where we observed that 38% of mice were diabetic by 2 weeks post CVB4 infection (Figure 4A, shaded region). In contrast, only 7% of C10-treated NOD mice infected with CVB4 were diabetic by that time (Figure 4A, shaded region), indicating that C10 effectively reduced CVB4 acceleration of T1DM in NOD mice. NOD mice not treated with virus but treated daily with sham, DMSO, or C10 injections were also included in this study, however no animals in any of these groups were diabetic by 10 weeks of age (Figure 4A, dashed lines). After the initial CVB4-induced spike in diabetes within the first 2 weeks (viral acceleration phase), we observed a plateau in diabetes development in all virus infected groups which preceded a subsequent gradual increase in diabetes development (Figure 4A). This plateau effect is in agreement with previous observations [55], and reflects what we believe to be the period of prolonged protection from disease expression mentioned above which is conferred by CVB4 in mice that did not have a “critical threshold” of insulitis present at the time of infection [55]. The gradual increase in incidence of diabetes following this plateau is likely due to the genetic predisposition of NOD mice to spontaneously develop T1DM at this age [55]. The pattern of diabetes development in non-virus-infected NOD mice indicates the spontaneous development of T1DM that is observed in this genetically predisposed strain of mice. It is noteworthy that we observed no effect of C10 on the spontaneous (genetically determined component) development of T1DM in the NOD mouse, which supports our hypothesis that C10’s protective effects are specific to molecular pathways involved in the environmental induction/acceleration of disease expression.

It is also noteworthy to mention that we observed no apparent toxic effects in the mice treated with C10. NOD mice treated with C10 for an extended period of time (16 weeks) show no signs of toxicity related to the C10 treatment [*i.e*., C10-treated animals are comparable in weight to control-treated animals (Figure 4B) and showed no additional signs of distress (*i.e*., cachexia, poor grooming, absence of normal dietary patterns)] throughout the duration of the experiment. In addition, we observed no beta cell toxicity of C10 in any of our cell culture experiments described herein, nor have we seen toxicity of C10 in any of our previously published *in vitro* or* in vivo* studies [37,38,39,40,56,57]. Together, this suggests toxicity associated with C10 is minimal and that C10 is likely to be as well tolerated or possibly better tolerated than its parent compound methimazole.

## 3. Experimental

### 3.1. Reagents

C10 was provided by the Interthyr Corporation (Marietta, OH, USA). A 200 mM stock solution of C10 was dissolved in dimethylsulfoxide (DMSO) (Sigma, St. Louis, MO, USA), which was further diluted to 0.5 mM for*in vitro* experiments in culture medium. pIC was purchased from Sigma (St. Louis, MO, USA). Edward’s CVB4 was kindly provided by Dr. Roger Loria, Virginia Commonwealth University.

### 3.2. Cell Culture and Transfections

NIT-1 and TC-6 beta cell lines were purchased from American Type Culture Collection (ATCC) (Manassas, VA, USA). NIT-1 cells were cultured in Ham's F12K medium (ATCC) supplemented with 10% heat-inactivated dialyzed fetal bovine growth serum (FBS, Gibco, Carlsbad, CA, USA). TC-6 cells were cultured in Dulbecco’s Modified Eagle Medium (Hyclone, Red Bank, NJ, USA) supplemented with 15% FBS (Hyclone). Cells were incubated at 37 °C in a humidified atmosphere of 5% CO_2_. Cells were transfected with pIC using Lipofectamine 2000 (LF2000) (Invitrogen, Carlsbad, CA, USA). Serum-free media with liposome complexes was replaced at 24 h post-transfection with appropriate cell culture media for experiments lasting longer than 24 h.

### 3.3. Cellular Viability Assays

Cells were seeded in triplicate at a density of 2.5 × 10^4^ cells per well. The number of viable cells was quantified with the Cell Titer-Glo Luminescent Cell Viability Assay (Promega, Madison, WI, USA). Luminescence was measured with the Lumat LB 9507 luminometer (Berthold Technologies, Oak Ridge, TN, USA). A standard curve was generated to correlate cell numbers with luminescence activity.

### 3.4. Quantitative Real Time PCR

Total RNA was isolated (RNeasy Kit, Qiagen, Valencia, CA, USA), treated with DNase (RNase-Free DNAse Kit, Qiagen), and quantified with the RNA 6000 Nano Chip (Agilent Technologies, Santa Clara, CA, USA) and the 2100 Bioanalyzer (Agilent Technologies). cDNA was synthesized using the High Capacity cDNA Reverse Transcription Kit (Applied Biosystems, Carlsbad, CA, USA). Pre-amplification of cDNA was done using the TaqMan PreAmp Master Mix Kit (Applied Biosystems, Carlsbad, CA, USA). Real-time PCR was performed using murine CXCL10, IFNβ, TNFα, TLR3, and MHC Class I Gene Expression Assays (Applied Biosystems). Non-preamplified cDNA was used for CXCL10 real-time PCR, whereas pre-amplified cDNA was used to amplify IFNβ, TNFα, TLR3, and MHC Class I by real-time PCR. Changes in gene expression were calculated using the ΔΔCt method.

### 3.5. Quantitative Real Time PCR Arrays

TC-6 and NIT-1 cells were transfected with pIC in the presence or absence of C10 or DMSO (solvent) for 24 h. Total RNA was isolated (RNeasy MiniElute Cleanup Kit, Qiagen) and quantified with the RNA 6000 Nano Chip (Agilent Technologies) and the 2100 Bioanalyzer (Agilent Technologies). cDNA was synthesized using the RT² First Strand Kit (SABiosciences, Frederick, MD, USA). Real-time PCR was performed with the Mouse Toll-Like Receptor Signaling Pathway PCR Array (SABiosciences) and the Mouse Inflammatory Cytokines and Receptors PCR Array (SABiosciences) using the Mx3000P QPCR system (Stratagene, La Jolla, CA, USA). Fold changes in gene expression were calculated using the ΔΔCt method. Statistical analysis was performed using the two-tailed paired Student’s *t*-test, and a *p*-value of less than 0.05 was considered significant.

### 3.6. CXCL10 ELISA

CXCL10 protein levels were measured in the cell culture supernatants using the mouse CXCL10/IP-10 Quantikine ELISA kit according to the manufacturer’s instructions (R&D Systems, Minneapolis, MN, USA).

### 3.7. NOD Mouse Experiment

Mice were housed in a sterile/pathogen free facility. Seven week old wild-type NOD female mice were obtained from Jackson Labs (Bar Harbor, ME, USA) and allowed to acclimate for 1 week prior to initiation of studies. Eight week old NOD mice were infected by intraperitoneal (i/p) injection with 5 × 10^5^ plaque forming units of CVB4 [58]. Indicated animals received daily i/p sham injections and/or daily injections of DMSO (control solvent) or 1 mg/kg C10 following CVB4 infection. Non-infected control mice received similar sham, DMSO, or C10 treatments. Weight and blood glucose were measured weekly. Diabetes was defined as blood glucose values exceeding 240 mg/dL on consecutive days (at least 24 h apart) and confirmed by urinary glucose excretion (Diastix, Bayer, Pittsburgh, PA, USA).

## 4. Conclusions

The results described herein indicate that C10 significantly protects beta cells from dsRNA-induced cytotoxicity and up-regulation of cytokines involved in beta cell destruction in beta cells in culture. Moreover, we report the novel finding that C10 protects NOD mice from CVB4-induced acceleration of T1DM. Together, these results demonstrate that C10: (1) possesses novel beta cell protective activity from dsRNA induction of apoptosis and inflammation; (2) protects genetically predisposed NOD mice from CVB4-induced acceleration of T1DM, suggesting that C10 may have potential clinical relevance for T1DM; and (3) may be a useful tool to glean a greater understanding of the role that dsRNA-mediated responses play in the pathogenesis of T1DM.
